# Supplementary material for: Mechanisms governing the pioneering and redistribution capabilities of the non-classical pioneer PU.1
Source: Nat Commun. 2020 Jan 21;11:402. doi: 10.1038/s41467-019-13960-2 (PMC6972792; doi:10.1038/s41467-019-13960-2)
Supplement: Supplementary file 7 — Source data [file 41467_2019_13960_MOESM7_ESM.zip › Source_Data/FigureS2/FigureS2B_MotifScanOutput/KmeansCluster7/homerResults/motif17.similar.html]

motif17

## Information for motif17

C
G
T
A
T
C
A
G
G
C
A
T
A
C
G
T
G
A
T
C
G
A
C
T
A
G
T
C
T
C
G
A
A
G
C
T
T
C
A
G
T
C
G
A
A
C
T
G
C
T
G
A
  
Reverse Opposite:  

A
G
C
T
T
G
A
C
A
G
C
T
A
G
T
C
T
C
G
A
A
G
C
T
T
C
A
G
C
T
G
A
C
A
T
G
C
G
T
A
C
G
T
A
A
G
T
C
G
C
A
T
  

|  |  |
| --- | --- |
| p-value: | 1e-64 |
| log p-value: | -1.474e+02 |
| Information Content per bp: | 1.744 |
| Number of Target Sequences with motif | 130.0 |
| Percentage of Target Sequences with motif | 4.05% |
| Number of Background Sequences with motif | 198.8 |
| Percentage of Background Sequences with motif | 0.45% |
| Average Position of motif in Targets | 101.4 +/- 42.0bp |
| Average Position of motif in Background | 99.2 +/- 78.1bp |
| Strand Bias (log2 ratio + to - strand density) | 0.1 |
| Multiplicity (# of sites on avg that occur together) | 1.22 |
| Motif File: | file (matrix) reverse opposite |

### Similar de novo motifs found

|  |  |  |  |  |  |  |  |
| --- | --- | --- | --- | --- | --- | --- | --- |
| Rank | Match Score | Redundant Motif | P-value | log P-value | % of Targets | % of Background | Motif file |
| 1 | 0.954 | C T A G G C A T A C G T T A G C A G C T G A T C T C G A G A C T T C A G T C G A A T C G C T G A | 1e-63 | -145.191866 | 4.33% | 0.55% | motif file (matrix) |
| 2 | 0.947 | C T G A T A C G C G A T A C G T A G T C G A C T A G T C G T C A G A C T T C A G C T G A A C T G T C G A C G A T | 1e-62 | -142.925293 | 3.68% | 0.37% | motif file (matrix) |
| 3 | 0.755 | G T A C C T G A A G T C T A C G T C G A A T C G G C T A G C A T T A G C C G A T | 1e-36 | -85.003638 | 3.58% | 0.72% | motif file (matrix) |
